# Efficient Solketal Synthesis via Acetalization Using Sulfonated Carbon From Lavender Leaf Waste After Essential Oil Extraction

**DOI:** 10.1002/cbdv.71558

**Published:** 2026-08-08

**Authors:** Julianne A. Bruno, Guilherme R. Pereira, Kelly R. Francisco, Gabriel H. M. de O. Pinto, Eraldo Lima, Ulisses A. Pereira, Garbas A. dos S. Junior, Patrícia F. Pinheiro

**Affiliations:** ^1^ Department of Chemistry Federal University of Viçosa Viçosa Minas Gerais Brazil; ^2^ Department of Natural Sciences Mathematics and Education Federal University of São Carlos Araras São Paulo Brazil; ^3^ Department of Entomology ‐ BIOAGRO Federal University of Viçosa Viçosa Minas Gerais Brazil; ^4^ Institute of Agricultural Sciences Federal University of Minas Gerais Montes Claros Minas Gerais Brazil

**Keywords:** (2,2‐dimethyl‐1,3‐dioxolan‐4‐yl)methanol, biomass residue, catalysis, lavender waste, sulfonated carbon

## Abstract

Biomass‐based catalyst research is advantageous for the biodiesel industry's sustainability due to the catalysts' readily available raw materials, nontoxic properties, and biodegradability. In this study, sulfonated carbon catalysts were synthesized from lavender biomass residue, obtained after essential oil extraction, through hydrothermal carbonization (HTC) followed by in situ sulfonation, and applied in the acetalization of glycerol with acetone to produce solketal, a bio‐based fuel additive. To the best of our knowledge, this type of residue has not yet been explored for this specific application. Characterization by XRD and Fourier‐transform infrared spectroscopy (FT‐IR) confirmed the amorphous carbon structure and successful incorporation of ─SO_3_H groups. The formation of solketal was confirmed by nuclear magnetic resonance (NMR) and gas chromatography–mass spectrometry (GC–MS). The catalyst exhibited excellent performance under mild conditions, achieving 97% glycerol conversion and 98% selectivity toward solketal. Optimal conditions (40°C, 30 min, 7.5 wt% catalyst loading, and a glycerol‐to‐acetone molar ratio of 1:20) afforded high glycerol conversion and selectivity toward solketal. The catalyst exhibited remarkable stability, maintaining >96% conversion and >97% selectivity after seven reuse cycles, confirming the potential of lavender waste as a sustainable precursor for catalyst development. These results highlight the potential of lavender agro‐residues as a sustainable carbon source for the production of efficient heterogeneous catalysts in renewable fuel applications.

## Graphical abstract

Sulfonated carbon catalysts were synthesized from lavender leaf waste after essential oil extraction via hydrothermal carbonization (HTC). Under mild, low‐energy conditions, the catalyst achieved 97% glycerol conversion and 98% solketal selectivity. This approach valorizes agricultural residues and crude glycerol from biodiesel production, contributing to sustainable waste conversion and circular biorefinery strategies.

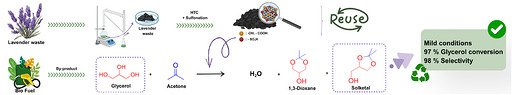

## 1 Introduction

The growing environmental concerns associated with the intensive use of fossil resources and the consequent increase in greenhouse gas emissions have driven the search for more sustainable chemical processes. In this context, biomass has emerged as a renewable and abundant carbon source capable of replacing fossil‐based feedstocks, playing a key role in the transition toward a circular and low‐carbon economy [1, 2]. In this context, the development of sustainable processes based on renewable resources is essential to support global efforts toward carbon neutrality and climate change mitigation.

Lavender comprises over 39 species [3], with *Lavandula angustifolia*, *Lavandula spica*, and *Lavandula hybrida* being the main sources of essential oil production [4]. Among these, *L. angustifolia* stands out due to its high commercial value and broad applications. However, its low essential oil content (0.8%–1.3%) leads to the generation of large amounts of biomass waste during distillation [5]. This residual biomass represents a promising feedstock for valorization, including applications in construction materials, soil conditioning, and as a precursor for biochar [6, 7, 8, 9].

Managing this waste represents a global challenge [10]. A promising alternative is its valorization for the synthesis of heterogeneous sulfonated carbon catalysts [11], which exhibit high thermal stability and resistance under acidic and alkaline conditions [12]. While traditionally derived from carbohydrate‐based precursors, these materials are increasingly produced from biomass as a sustainable and abundant resource [12]. Although various biomass‐derived catalysts have been reported [13], the use of residues from essential oil extraction remains limited [14]. Their utilization adds value to this waste stream and aligns with circular economy principles.

The transesterification of vegetable oils or animal fats for biodiesel production generates glycerol as the main by‐product, making it an important platform molecule for chemical valorization. It is estimated that by 2025, global biodiesel production will generate approximately 2.3 billion liters of glycerol. Its acetalization with acetone has been widely studied as an efficient route for solketal synthesis [15, 16, 17]. Solketal is used as a solvent, plasticizer, pharmaceutical agent [17], fuel additive, and antifreeze [18, 19]. However, conventional homogeneous catalysts, such as H_3_PO_4_, HCl, and PTSA, present limitations including corrosiveness and poor recoverability [20]. In contrast, heterogeneous catalysts offer advantages such as easy separation and reusability [21, 22]. Various systems have been explored, including heteropolyacids [23] and zeolites [24].

Recent studies have reported biomass‐derived carbonaceous catalysts for this reaction. Kowalska‐Kuś et al. [25] used catalysts obtained from used tires, while Das et al. [26] developed a catalyst from pine cone biomass (*Pinus kesiya*). Other sources include pearl millet cobs [27], red macroalgae [28], and lotus leaves [29]. Despite these advances, residues from essential oil extraction remain underexplored, highlighting the potential of lavender waste as a sustainable precursor.

In this study, a heterogeneous acid catalyst was synthesized from lavender biomass residues obtained after essential oil extraction via hydrothermal carbonization (HTC) with in situ functionalization. The catalyst's performance was systematically evaluated to optimize glycerol conversion and solketal selectivity. This work integrates the use of biomass as a catalyst precursor with the production of potential biofuels, contributing to the development of sustainable chemical processes, biomass valorization strategies, and waste utilization. By bridging waste valorization and catalytic efficiency, this study highlights the potential of circular economy approaches for transforming low‐value residues into high‐performance materials, opening new avenues for the design of sustainable biomass‐derived catalysts and their application in green chemical processes.

## 2 Experimental Section

### 2.1 Materials

Solketal was synthesized from glycerol (99.5%) and acetone (99.5%), which were acquired from Sciavicco. Sulfuric acid and hydrochloric acid, both of analytical grade, used in the catalyst synthesis, were purchased from Êxodo Científica and Synth, respectively. Deuterated chloroform used for spectral acquisition was obtained from Sigma‐Aldrich, all with a purity greater than 98%. All reagents were used without additional purification.

### 2.2 Catalyst Synthesis

**SCHEME 1:**
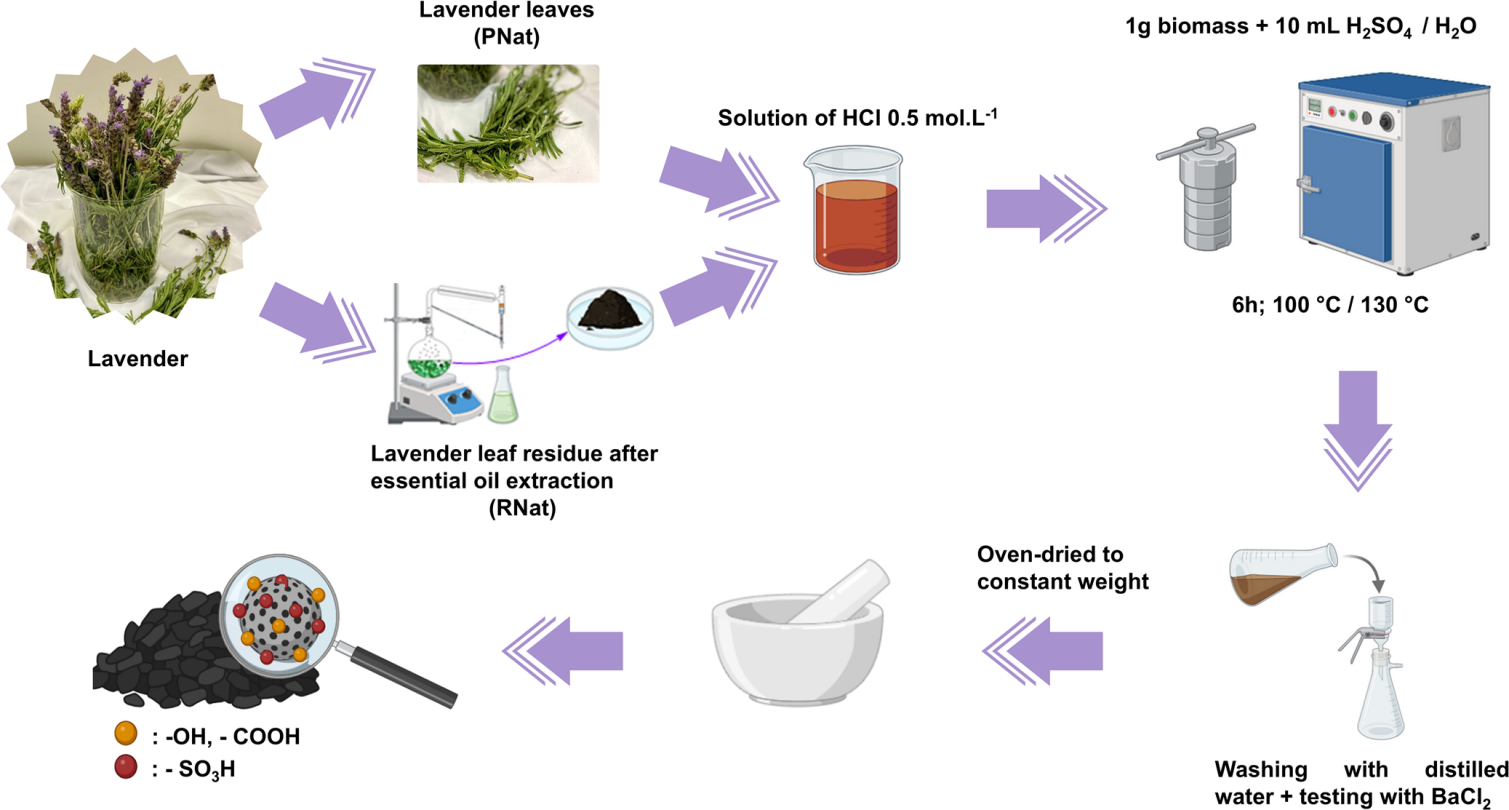
Schematic representation of the preparation of sulfonated carbonaceous materials from lavender leaves (PNat) and lavender leaf residue after essential oil extraction (RNat), including HCl treatment, HTC, and sulfonation.

The plant material used in this study was identified as *Lavandula dentata* L. (Lamiaceae) by Dr. Andreia Fonseca Silva. A voucher specimen was prepared and deposited in the PAMG Herbarium under accession number PAMG 59821. The material was collected in Viçosa, Minas Gerais, Brazil, on May 2, 2025. After collection, a portion of the leaves was used directly for the synthesis of sulfonated catalysts via HTC with in situ functionalization, whereas the remaining fraction was subjected to hydrodistillation for essential oil extraction prior to catalyst preparation. The extraction, performed in triplicate, generated a residue (RNat). Both the residue and the leaves were washed with distilled water, dried in an oven at 80°C for 72 h, ground, and treated with HCl 0.5 mol L^−1^ for 72 h (biomass: HCl 1:3, w v^−1^). The HCl‐treated biomass (RHCl/PHCl) was then washed to pH 7 and dried in an oven at 80°C until a constant weight was achieved [30]. For hydrothermal treatment and sulfonation of the lavender biomass, 1 g of biomass was mixed with 10 mL of concentrated H_2_SO_4_, stirred for 0.5 h at room temperature, and then transferred to an autoclave and heated at 100°C or 130°C in an oven for 6 h (Scheme 1). The resulting black solid was washed until sulfate‐free (tested with a BaCl_2_ aqueous solution), filtered, and dried at 80°C in an oven for 24 h, yielding the sulfonated catalyst [30]. For catalyst synthesis, two HTC temperatures (100°C and 130°C) were selected to investigate the effect of carbonization severity on the physicochemical properties, acid site density, and catalytic performance of the resulting materials. The lower temperature was evaluated as a less energy‐intensive alternative, while 130°C was chosen based on previous studies that demonstrated its suitability for the preparation of acidic carbonaceous materials [14]. Temperatures within this range have been reported to promote the formation of partially carbonized carbon frameworks while preserving oxygen‐containing functional groups, which are important for subsequent sulfonation and catalytic activity.

The biomass samples were ground using a mortar and pestle until a fine powder was obtained. The sulfonated materials obtained at 100°C and 130°C were designated as RSul100 and RSul130, respectively. The non‐functionalized material was also prepared at both temperatures (RBr100 and RBr130) using water instead of H_2_SO_4_. A similar process was applied to the leaves without essential oil extraction, resulting in the following designations: PNat (washed/dried leaves), PHCl (HCl‐treated), PBr100 (hydrothermal with water at 100°C), PBr130 (hydrothermal with water at 130°C), PSul100 (sulfonated catalyst at 100°C), and PSul130 (sulfonated catalyst at 130°C). Table 1 summarizes the materials obtained, from the precursor biomass to the sulfonated carbonaceous catalysts, along with their assigned codes.

**TABLE 1:** Description of lavender‐derived samples and treatments applied.

Code	Description
RNat	Residue from lavender leaves after essential oil extraction (hydrodistillation)
RHCl	RNat treated with HCl (biomass:HCl 1:3, w v^−1^), washed to pH 7, and oven‐dried
RBr100	Hydrothermally carbonized RHCl at 100°C (without sulfonation, water used instead of H_2_SO_4_)
RBr130	Hydrothermally carbonized RHCl at 130°C (without sulfonation, water used instead of H_2_SO_4_)
RSul100	Sulfonated catalyst from RHCl, hydrothermal treatment at 100°C with H_2_SO_4_
RSul130	Sulfonated catalyst from RHCl, hydrothermal treatment at 130°C with H_2_SO_4_
PNat	Lavender leaves (without oil extraction), washed and oven‐dried
PHCl	PNat treated with HCl (biomass:HCl 1:3 w v^−1^), washed to pH 7, and oven‐dried
PBr100	Hydrothermally carbonized PHCl at 100°C (without sulfonation)
PBr130	Hydrothermally carbonized PHCl at 130°C (without sulfonation)
PSul100	Sulfonated catalyst from PHCl, hydrothermal treatment at 100°C with H_2_SO_4_
PSul130	Sulfonated catalyst from PHCl, hydrothermal treatment at 130°C with H_2_SO_4_

### 2.3 Catalyst Characterization

Various characterization techniques were employed to analyze the physicochemical properties of the synthesized catalyst. The obtained catalysts were analyzed using an x‐ray diffractometer (XRD) (D8 Discover (DaVinci), Bruker). The equipment operated at a voltage of 40 kV, utilizing a Cu 2Kα radiation source, and the samples were scanned over a 2*θ* range of 10°–70°. The surface morphology of these catalysts was analyzed by scanning electron microscopy (SEM) (JSM‐6010LA, JEOL) after gold metallization using a sputter coater (Quorum, Q150R S). The morphological characterization of the samples was conducted using transmission electron microscopy (TEM) with a TALOS Cold FEG (Thermo Fisher Scientific) operating at 200 kV, as well as a TECNAI G2 F20 HRTEM (FEI Company), also operated at 200 kV. Sample preparation involved dilution and deposition of the solution onto copper grids coated with a carbon film. Fourier‐transform infrared spectroscopy (FT‐IR) was obtained in a spectrophotometer (ALPHAA II, BRUKER, USA) equipped with an attenuated total reflectance accessory (ATR) over the region of 400–4000 cm^−1^ with 16 scans and 4 cm^−1^ of spectral resolution. Thermogravimetric analysis (TGA) (STA 6000, PerkinElmer) was carried out from room temperature up to 600°C, with a heating rate of 10°C min^−1^ under a nitrogen atmosphere. Nitrogen adsorption–desorption isotherms were obtained for RSul100 using a Nova 600 series instrument (Anton Paar). Elemental analysis (CHNS) was performed using a simultaneous elemental analyzer (TruSpec Micro, LECO), with calibration based on the LECO Cystine standard, which contains: N 11.64%, C 29.98%, H 5.02%, and S 26.71%. Proton nuclear magnetic resonance (^1^H NMR) spectra and carbon nuclear magnetic resonance (^13^C NMR) spectra (AvanceCore, Bruker 400 MHz) were performed using a 5 mm broadband inverse detection (BBI) probe, equipped with ATMA, a field gradient generator unit, and temperature control. Gas chromatography–mass spectrometry (GC–MS) analyses were performed using a QP2010SE instrument (Shimadzu, Japan). Helium was used as the carrier gas at a flow rate of 1.80 mL min^−1^ and a linear velocity of 48.4 cm s^−1^. The injector temperature was maintained at 220°C. A fused silica capillary column (30 m × 0.25 mm i.d.) coated with an Rtx‐5MS stationary phase (film thickness, 0.25 µm) was used. The acidic strength of the material was estimated by potentiometric titration using a Metrohm 904 Titrando system equipped with a Metrohm 803 Ti Stand (Metrohm, Switzerland) [31]. Typically, the material (50 mg) was suspended in acetonitrile (CH_3_CN, 30 mL) and was magnetically stirred for 15 h at room temperature. After, the content was titrated with a solution of *n*‐butylamine in acetonitrile (0.025 mol L^−1^). A 50 mg portion of the material was added to a standardized 0.1 mol L^−1^ NaOH solution and stirred for 3 h. The mixture was then filtered through filter paper to remove the solid residue. Subsequently, 10 mL aliquots of the filtrate were taken in triplicate, phenolphthalein was added as an indicator, and the solution was titrated with a standardized 0.1 mol L^−1^ HCl solution. The total acidity was calculated using Equation (1) below, and the results were expressed as mol H^+^ g^−1^ of catalyst [14].

(1) $$C\;\left(H^{+}\right)=\frac{\left(V_{n}-V_{t}\right)\;x\;C_{\mathrm{HCl}}}{m}$$

where: $V_{n}$ is the volume (L) of HCl used to neutralize 10.00 mL of NaOH in the blank; $V_{t}$ is the volume (L) of HCl used in the titration of 10.00 mL of the NaOH solution that was stirred with the carbonaceous material; $C_{\mathrm{HCl}}$ is the concentration (mol L^−1^) of the HCl solution; m is the mass of the carbonaceous material sample (g).

### 2.4 Acetalization of Glycerol Using Catalysts Derived From Lavender Biomass

**SCHEME 2:**
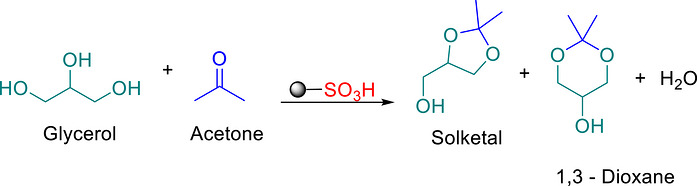
Acetalization of glycerol using lavender biomass as a solid catalyst (●─SO_3_H).

The catalysts synthesized were used in the acetalization of glycerol (Scheme 2). Glycerol, acetone, and the catalyst were added to a 10 mL flask and stirred at room temperature for 6 h. A preliminary catalytic screening was performed for all synthesized materials under identical conditions (room temperature, glycerol‐to‐acetone molar ratio of 1:20, 5 wt% catalyst (based on the mass of glycerol), and 6 h reaction time), while a blank experiment was conducted without catalyst for 24 h. The screening results (Figure S1) identified RSul100 as the most active catalyst, which was subsequently selected for characterization and reaction optimization. Based on the initial tests, 2 h was established as the maximum reaction time for optimization studies, and the optimal catalytic performance was achieved after 30 min under the optimized conditions.

The reaction parameters studied were substrate ratio (1:5–1:25), catalyst loading (1–10 wt%), temperature (40°C–80°C), and reaction time (15–120 min). The optimized conditions were identified and established as a substrate ratio of 1:20 {glycerol 3.62 mmol (0.333 g), acetone 72.4 mmol (4.20 g)}, catalyst loading of 7.5 wt% (0.025 g), a temperature of 40°C, and a reaction time of 30 min. Upon completion, the reaction mixture was filtered, and the catalyst was recovered for reuse. The filtered solution was then concentrated using a rotary evaporator to remove excess acetone. All parameters were evaluated in triplicate.

The solketal product was studied using gas chromatography and NMR. Glycerol conversion and the selectivity between solketal (the 5‐membered cyclic solketal) and acetal (the corresponding 6‐membered cyclic acetal) were verified from GC–MS with the help of Equations (2) and (3).

(2) $$\mathrm{Glycerol}\;\mathrm{conversion}\;\left(\%\right)=\;\;\frac{Mol\;glycerol\;converted}{Initial\;mol\;glycerol}\times 100$$

(3) $$\mathrm{Solketal}\;\mathrm{selectivity}\;\left(\%\right)=\;\;\;\frac{Mol\;solketal\;formed}{Mol\;glycerol\;converted}\;\times 100$$

Glycerol and the synthesized solketal were analyzed by FT‐IR (Figure S2) to confirm product formation and to compare the spectra with previously reported data [32].

### 2.5 Catalyst Recycling

After completion of the reaction, the mixture was centrifuged to separate the catalyst from the solution. The supernatant was discarded, and the catalyst was washed with ethanol, repeating the procedure four times. The recovered catalyst was then dried in an oven at 80°C for 5 h. This step was repeated four additional times under the optimized reaction conditions (glycerol/acetone molar ratio of 1:20, 7.5 wt% catalyst loading, 40°C, and 15 min) to evaluate the catalyst's reusability. All cycles were conducted in triplicate.

## 3 Results and Discussion

Eight catalysts were synthesized in this study: four derived from lavender leaves (PSul100, PBr100, PSul130, and PBr130) and four from lavender leaf residue remaining after essential oil extraction (RSul100, RBr100, RSul130, and RBr130). Two carbonization/sulfonation temperatures, 100°C and 130°C, were evaluated, with all reactions conducted for 6 h.

The catalysts were evaluated in a model acetalization reaction between glycerol and acetone at room temperature for 6 h. Aliquots were collected at 1‐h intervals to determine the minimum reaction time required and to identify the catalyst that provided the highest glycerol conversion and solketal selectivity, favoring the formation of the five‐membered ring isomer (Figure S1). In the absence of a catalyst, the acetalization reaction requires more than 24 h to proceed and still yields unsatisfactory results, with glycerol conversions below 20%, as indicated by the white‐coded data in Figure S1. Among the tested catalysts, RSul100 exhibited the best performance in terms of both glycerol conversion and selectivity toward solketal. Therefore, RSul100 was selected for further characterization and for subsequent optimization of the acetalization reaction.

### 3.1 Characterization of the Synthesized Catalyst

**FIGURE 1:**
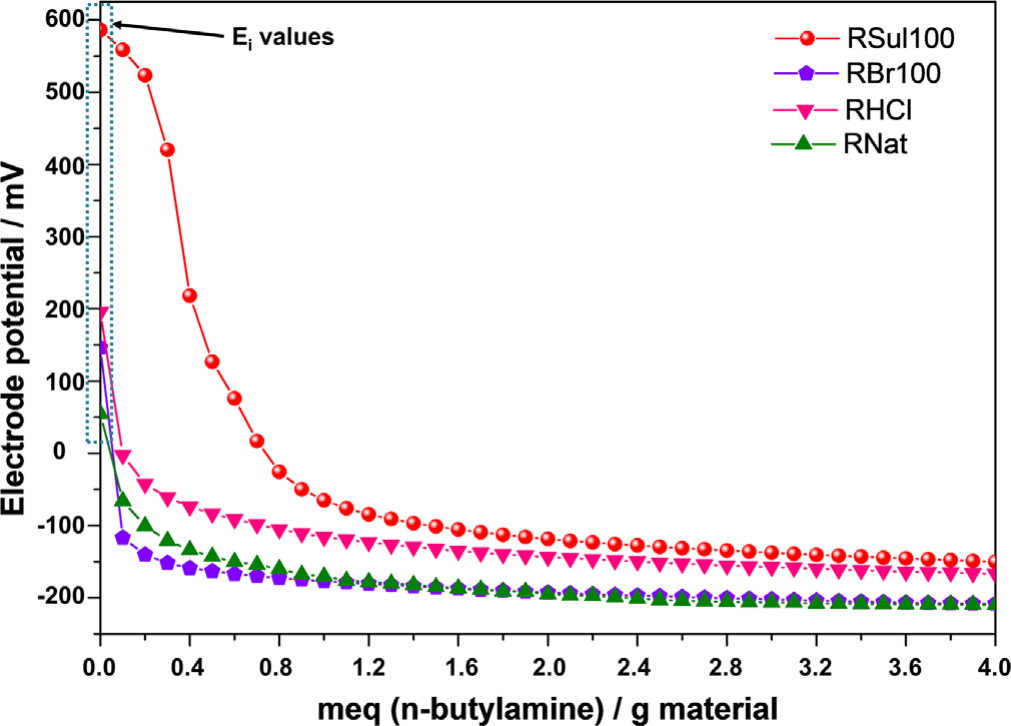
Potentiometric titration profiles of the materials with *n*‐butylamine: RSul100 (red), RBr100 (purple), RHCl (pink), and RNat (green). The initial electrode potential (*E*_i_) values indicate the relative acid strength of the catalysts.

The first analysis performed was potentiometric titration with *n*‐butylamine, employed as one of the methods to evaluate the surface acidity of solid materials. The maximum acid strength of the catalyst surface is indicated by the initial electrode potential value, *E*_i_ (mV). According to the criteria proposed by Pizzio et al. [31], the acid strength of a catalyst can be classified as very strong (*E*_i_ > 100 mV), strong (0< *E*_i_ < 100 mV), weak (–100< *E*_i_ < 0 mV), and very weak (*E*_i_ < –100 mV). Figure 1 presents the potentiometric titration curves of the sulfonated carbon catalyst that showed the best performance in the acetalization of glycerol, along with its precursor material. Based on Pizzio's classification, the RSul100, RBr100, and RHCl samples exhibit very strong acid sites, since their initial potentials are above 100 mV, namely, 585.9, 146.4, and 195.7 mV, respectively. In contrast, the precursor material RNat exhibits only strong acid sites, with *E*_i_ = 54.8 mV, which falls within the 0–100 mV range, consistent with the presence of natural acidic groups on its surface. Notably, the *E*_i_ value of RSul100 is comparable to that of typical strong acids, such as H_2_SO_4_, *p*‐toluenesulfonic acid, and Keggin‐type heteropolyacids (H_3_PW_12_O_40_, H_3_PMo_12_O_40_, and H_4_SiW_12_O_40_), which typically show *E*_i_ values between 560 and 580 mV [33, 34, 35].

Additionally, the total density of acid sites for all samples was determined by acid–base back‐titration (Table 2) [36, 37]. Among the investigated materials, the sulfonated catalyst RSul100 exhibited the highest total acid density, reaching 0.99 ± 0.07 mmol H^+^ g^−1^, thereby confirming the effective incorporation of ─SO_3_H groups during the in situ sulfonation process. In contrast, the non‐sulfonated hydrothermal carbon (RBr100) and the untreated natural residue (RNat) exhibited significantly lower acid densities, corresponding to 0.45 ± 0.09 and 0.34 ± 0.10 mmol H^+^ g^−1^, respectively.

**TABLE 2:** Total acidity of the prepared materials determined by acid–base back‐titration^a^.

Sample	Total acidity (mmol H^+^. g^−1^)	Sample	Total acidity (mmol H^+^. g^−1^)
RSul100	0.99 ± 0.07	PSul100	0.92 ± 0.06
RSul130	0.90 ± 0.20	PSul130	0.89 ± 0.06
RBr100	0.45 ± 0.09	PBr100	0.51 ± 0.06
RBr130	0.40 ± 0.10	PBr130	0.38 ± 0.10
RNat	0.34 ± 0.10	PNat	0.30 ± 0.10

^a^ Total acidity values are expressed as mean ± standard deviation (*n* = 3).

A similar trend was observed for the materials obtained from the fresh plant, where the sulfonated samples (PSul100 and PSul130) exhibited higher acid densities than their non‐sulfonated counterparts (PBr100 and PBr130), further confirming the effectiveness of the sulfonation treatment in increasing the concentration of Brønsted acid sites. Furthermore, the materials synthesized at 100°C consistently exhibited higher acidity than those prepared at 130°C, indicating that lower carbonization temperatures favor the preservation and anchoring of ─SO_3_H groups within the carbon framework. This behavior is consistent with findings reported by Rechnia‐Gorący et al. [38] and Araujo et al. [39].

The non‐sulfonated materials RBr100, RBr130, PBr100, and PBr130 exhibited lower acid densities of 0.45 ± 0.09, 0.40 ± 0.10, 0.51 ± 0.06, and 0.38 ± 0.10 mmol H^+^ g^−1^, respectively, yielding relatively modest glycerol conversions (maximum values of 42%, 49%, 60%, and 50%, respectively). These results demonstrate that the catalytic performance is directly driven by the density of acid sites, particularly the ─SO_3_H groups introduced via sulfonation. The substantial increase observed in both acidity and catalytic conversion for RSul100 confirms that these Brønsted acid sites are the primary active centers driving the acetalization reaction.

Furthermore, the sulfonation process significantly increased the concentration of exchangeable protons within the carbonaceous matrix. Considering the metal‐free nature of the lavender‐derived carbon materials, these acidic sites are identified as Brønsted acid sites associated with ─SO_3_H functionalization [40, 41, 42]. A clear, direct correlation was observed between the Brønsted acid site density and glycerol conversion, confirming that these functional groups are the primary active species responsible for the superior catalytic efficiency of RSul100.

These findings are consistent with the potentiometric titration results, further supporting the presence of strong Brønsted acid sites in RSul100. The marked increase in acidity after sulfonation highlights the effectiveness of the hydrothermal treatment followed by sulfuric acid functionalization in generating active acid sites on the carbonaceous surface. This enhancement in acidity is directly correlated with the improved catalytic performance of the material.

Nitrogen adsorption–desorption analysis was performed to evaluate the textural properties of the synthesized catalyst, and the corresponding data are presented in the Supporting Information (Figure S3 and Table S1). The material exhibited a low BET surface area and a limited pore volume, indicating restricted accessible porosity; all the samples exhibit a typical type II isotherm of nonporous materials [41, 42, 43]. Therefore, the samples can be considered dense carbonaceous bulk materials with poorly developed porous networks, exhibiting minimal contributions from micro‐, meso‐, and macroporous structures within the carbon matrix [32, 42].

This behavior can be attributed to the relatively low carbonization temperature, which generally favors the formation of compact and partially condensed carbon structures with reduced nitrogen accessibility [32, 41, 42, 44, 45]. Furthermore, the incorporation of ─SO_3_H groups into the carbon framework may promote pore blocking and narrowing due to the high density of these functional groups anchored to the surface. Consequently, the presence of bulky sulfonic acid moieties, combined with structural densification under the acidic and thermal conditions employed during sulfonation, significantly reduces the accessible porosity of the material [37, 41, 46, 47].

Despite its limited textural properties, the catalyst exhibited outstanding efficiency in the acetalization of glycerol with acetone, demonstrating that catalytic activity is more strongly governed by the density of acid sites than by surface area alone [48]. Similar behavior has been reported for other biomass‐derived sulfonated carbon materials, where nonporous or low‐porosity frameworks integrated with high densities of ─SO_3_H groups play a dominant role in driving the catalytic performance. For instance, Filgueiras et al. [14] reported an exceptional 99% yield in the alcoholysis of furfuryl alcohol to ethyl levulinate using a structurally similar carbonaceous material, further supporting the hypothesis that surface functionalization can effectively compensate for restricted physical porosity. Likewise, Araujo et al. [48] synthesized a solid acid catalyst from açaí seed residues for the esterification of oleic acid with methanol, achieving a conversion of 95%.

This catalytic behavior can be explained by the high accessibility of reactants to the active acid sites. Nakajima and Hara [42] and Konwar, Mäki‐Arvela, and Mikkola [41] discussed that acidic carbonaceous materials, particularly those derived from partially carbonized and defect‐rich matrices, are capable of absorbing or retaining large amounts of hydrophilic molecules within the carbon structure. This is driven by the high density of oxygen‐containing functional groups (such as ─COOH and ─OH), in addition to the ─SO_3_H groups anchored to the flexible carbon framework. This characteristic substantially enhances the adsorption and accessibility of hydrophilic reactants to the active centers, thereby boosting the catalytic performance of these materials in various organic transformations [32, 41, 42].

**FIGURE 2:**
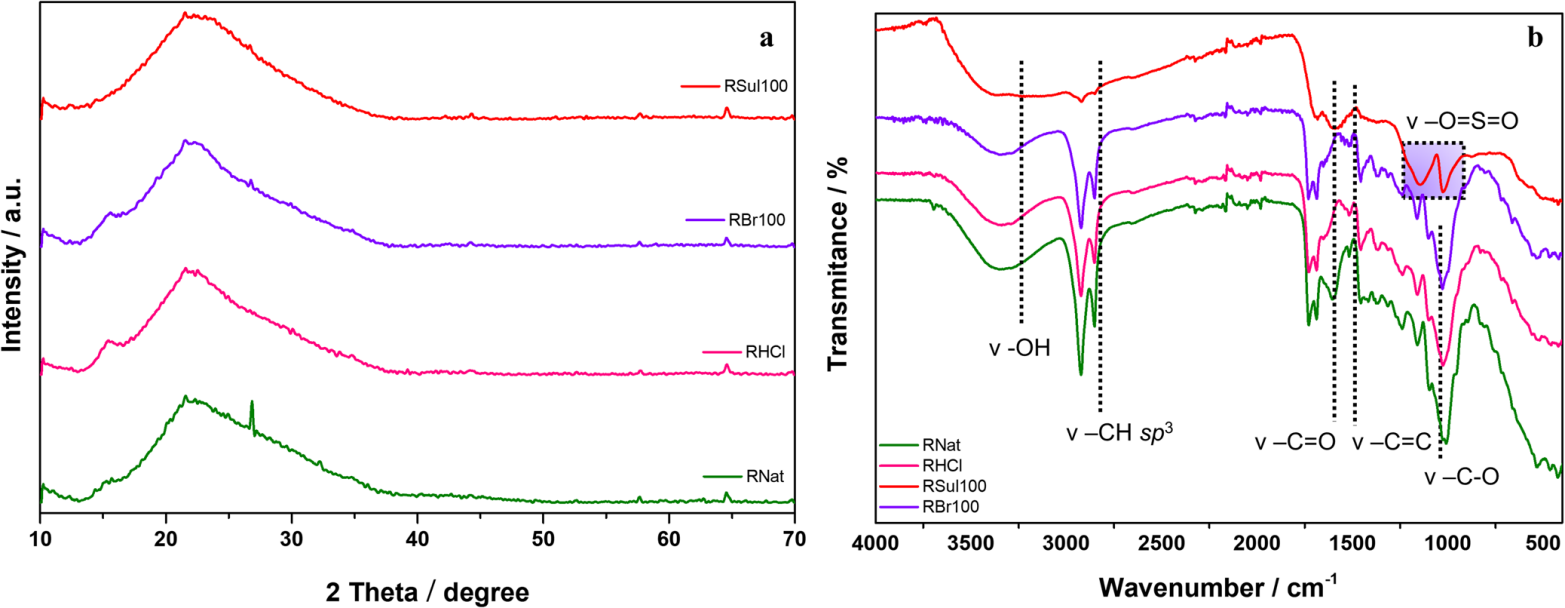
(a) Normalized XRD patterns of the materials at different stages of lavender residue processing: RSul100 (red), RBr100 (violet), RHCl (pink), RNat (green), and (b) FT‐IR spectra.

Since the catalytic performance is closely related to both surface chemistry and carbon structure, x‐ray diffraction (XRD) analysis was performed to investigate the structural organization of the synthesized materials. Figure 2a shows the XRD patterns of the sulfonated catalyst (RSul100), the raw lavender residue (RNat), the HCl‐washed material (RHCl), and the hydrothermally carbonized material without sulfonation (RBr100). The broad diffraction peaks observed in all diffractograms indicate the predominantly amorphous nature of the materials [32]. In all cases, a broad and weak diffraction peak corresponding to the C(002) plane was observed in the 2*θ* range of 10°–30°, which is characteristic of amorphous carbonaceous materials composed of polycyclic aromatic sheets arranged in a highly disordered manner [32].

FT‐IR was employed to identify the functional groups present in the studied materials and to confirm the occurrence of the sulfonation process. The results are presented in Figure 2b. In the precursor materials, RNat and RHCl, a broad absorption band at 3344 cm^−1^ is observed, corresponding to the O─H stretching vibration. Additionally, the bands centered at 2851 and 2920 cm^−1^ are attributed to the symmetric and asymmetric stretching vibrations of C─H and CH_2_ groups, respectively [32, 49]. A similar band pattern is also observed for the material RBr100. In all materials, absorption bands at 1601 cm^−1^ and within the range of 1685–1726 cm^−1^ are present, corresponding to the stretching vibrations of C═C and C═O bonds, respectively [32]. The presence of sulfonic acid groups in the catalyst was confirmed by the characteristic absorption peaks attributed to ─SO_3_H groups in the RSul100 sample: a symmetric stretching mode of the ─SO_2_ group at 1020 cm^−1^ and an asymmetric stretching mode at 1147 cm^−1^. These bands are absent in the precursor materials, confirming the successful sulfonation of the carbon surface [50, 51].

FT‐IR analysis confirmed the successful introduction of ─SO_3_H groups into the carbonaceous material derived from the residue. Moreover, a reduction in the intensity of the bands associated with aliphatic C─H vibrations was observed in the sulfonated sample. Taken together, these spectral changes indicate that the sulfonation process not only incorporated ─SO_3_H functionalities, but also induced structural rearrangement within the carbon framework [51, 52].

**FIGURE 3:**
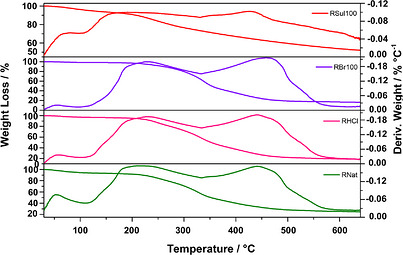
TGA and dTG thermograms of various stages of residue processing.

TGA was performed to evaluate the thermal stability of the catalyst. The TGA spectra, shown in Figure 3, reveal three distinct mass loss phases during heating for all materials.

The first event, observed at 80.9°C for the sulfonated material RSul100, is attributed to the elimination of physically adsorbed water or weakly bound acidic groups, resulting in a mass loss of 6.66% [53, 54]. In contrast, the non‐functionalized material RBr100 exhibited this event at 54.42°C, very similar to the starting material RNat at 53.5°C, with mass losses of 1.73% and 6.56%, respectively. Finally, the RHCl material showed this event at 58.99°C with a mass loss equivalent to 3.62% [54]. A higher mass loss rate is observed for the sulfonated material, which is attributed to increased hydrophilicity resulting from the incorporation of sulfonic groups [39].

The primary distinction among the samples, however, lies in the second and third events, which show significant variations in degradation rate and intensity. In the 190°C–350°C range (event 2), the RSul100 sample exhibited a mass loss of approximately 20.08%, attributed to the decomposition of surface functional groups such as ─OH, ─COOH, and especially sulfonic groups (─SO_3_H) [53]. The final event, occurring from 331.55°C to 610.68°C, corresponds to a 20.83% mass loss and is attributed to the partial decomposition of the carbon structure [39, 54]. For the non‐sulfonated samples, RBr100, RHCl, and RNat, the mass losses in the second event (190°C–330°C) were approximately 51.42%, 41.06%, and 40.40%, respectively. The mass losses in the third event (332.81°C–584.50°C) for these samples were 30.12%, 36.72%, and 28.08%, respectively. These losses can be attributed to the degradation of cellulose, hemicellulose, and lignin [54, 55, 56].

The results indicate that the sulfonated materials decompose at lower temperatures due to the presence of functional groups. In contrast, non‐functionalized materials and precursors show more significant mass losses at higher temperature ranges, which is associated with the degradation of their carbonaceous structure [57].

**FIGURE 4:**
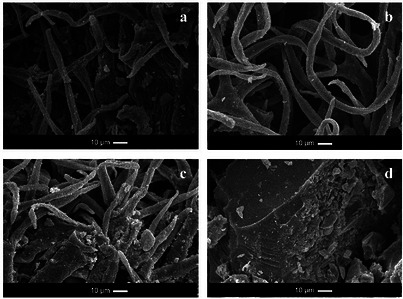
SEM images for (a) RNat, (b) RHCl, (c) RBr100, and (d) RSul100.

**FIGURE 5:**
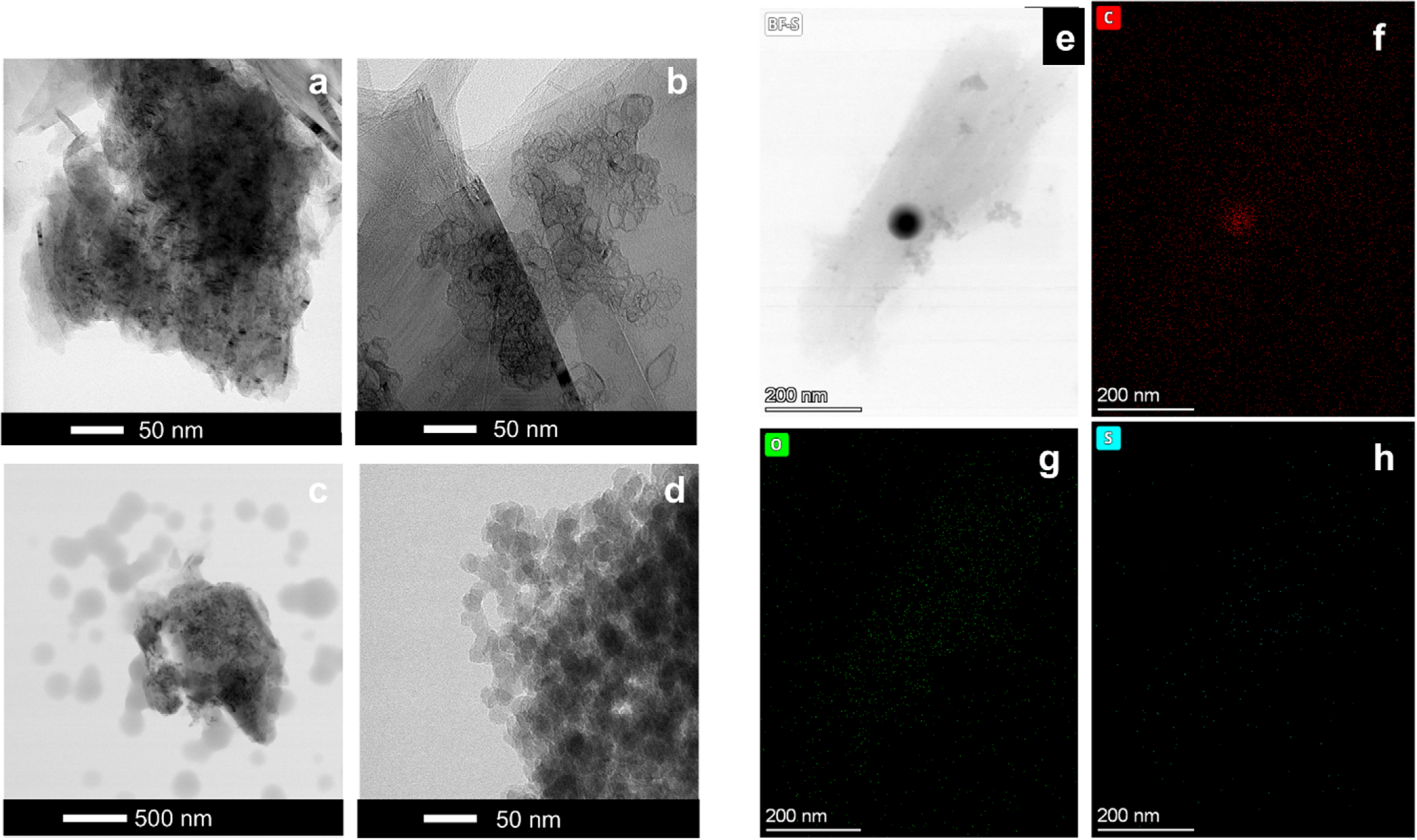
TEM images for (a) RNat, (b) RHCl, (c) RBr100, and (d) RSul100. Elemental mapping images: (e) TEM‐EDS image for sample RSul100, (f) C (red), (g) O (green), and (h) S (blue) in RSul100.

The morphology of the C─SO_3_H catalyst was evaluated using SEM, as shown in Figure 4, and TEM, as presented in Figure 5. Both analyses revealed significant morphological changes among the samples due to the applied treatments.

In the SEM analysis, the RNat sample (Figure 4a), which received no treatment, displayed a compact surface with low porosity, characteristic of the original fibrous lignocellulosic structure. The RHCl sample (Figure 4b), however, maintained its morphology relative to RNat, suggesting that acid washing effectively removes residual impurities without compromising the formed porous structure. Conversely, the RBr100 sample (Figure 4c), which underwent HTC, exhibited partial biomass degradation, forming cavities and irregular granules. These are characteristic signs of lignocellulosic matrix collapse. Finally, the RSul100 sample (Figure 4d), functionalized with sulfuric acid, showed a morphology with cavities and fissures, along with evidence of micro and mesoporous structures. These features are attributed to acid degradation and the introduction of sulfonated groups. The previously observed surface roughness and fibrous structure were no longer present, indicating dehydration of the cellulosic matrix, which aligns with our XRD results. The smoother, more compact surface can be attributed to the condensation of the carbonaceous structure, a result of carbonization and acid functionalization, which promotes the oxidation and degradation of the catalyst precursor [58, 59, 60].

TEM analysis revealed distinct morphological differences among the samples, consistent with the SEM observations. RNat and RHCl exhibited sheet‐like structures, whereas RBr100 and RSul100 displayed spherical amorphous aggregates, confirming the successful HTC process. TEM‐EDS analysis (Table 3) further indicated a higher sulfur content in the functionalized RSul100 compared to the other samples, despite the semi‐quantitative nature of this technique [32]. EDS elemental mapping images also verified the presence of carbon, oxygen, and sulfur in RSul100, supporting the successful incorporation of ─SO_3_H groups [61, 62]. Moreover, the homogeneous distribution of sulfur across the material surface suggests that the acidic sites are widely dispersed and readily accessible to the reactants, which likely contributes to the enhanced catalytic performance observed for RSul100 [61, 62, 63, 64].

**TABLE 3:** Elemental composition and morphological features of the carbonaceous materials determined by TEM‐EDS^a^

Sample	C (%)	O (%)	S (%)
RSul100	98.83	1.17	0.13
RBr100	95.20	4.80	0.03
RHCl	94.73	5.24	—
RNat	95.02	4.85	—

^a^ TEM‐EDS provides semi‐quantitative results as it probes only localized regions of the sample.

The final characterization was carried out by elemental analysis (CHNS) to determine the contents of carbon (C), hydrogen (H), nitrogen (N), and sulfur (S) in the samples. This analysis enabled the evaluation of chemical modifications induced by the treatment and functionalization processes. The results are summarized in Table 4.

**TABLE 4:** Elemental composition (CHNS) of the carbonaceous materials after different treatment and functionalization processes.

Sample	C (%)	H (%)	N (%)	S (%)
RSul100	50.61	4.75	1.40	6.96
RBr100	55.24	7.41	3.72	0.25
RHCl	49.10	6.85	3.11	0.87
RNat	47.50	6.41	2.44	ND^a^

^a^ Not Detected.

Comparing the CHNS results for RNat and RSul100, a reduction in volatile elements (H and N) is observed after the single‐step carbonization and sulfonation process [65]. An increase in both carbon and sulfur content was observed, indicating efficient carbonization and sulfonation of the material. The RSul100 and RBr100 samples, both of which underwent carbonization, showed an increase in carbon content due to the densification of carbon during the HTC process [66].

These data corroborate the complementary results obtained by other techniques, such as XRD and FT‐IR, confirming the efficiency of the applied chemical modification process.

### 3.2 Acetalization of Glycerol

After preparation and characterization of the lavender residue‐derived catalyst (C─SO_3_H), it was employed for solketal production via acetalization of glycerol with acetone. In a sealed tube, glycerol and acetone were mixed in a 1:20 molar ratio, followed by the addition of 5 wt% catalyst relative to glycerol. The reaction mixture was stirred at room temperature for 10 min prior to catalyst addition, and then the catalyst was added and the mixture stirred for 6 h at room temperature. Upon completion, the reaction mixture was filtered through filter paper and concentrated using a rotary evaporator to collect the product. Glycerol conversion was monitored by GC–MS. The product was compared with solketal reported in the literature [58]. Proton (^1^H) and carbon (^13^C) NMR spectroscopy confirmed the product as 2,2‐dimethyl‐1,3‐dioxolane‐4‐methanol (solketal, Figure S4) ^1^H NMR (400 MHz, CDCl_3_, 25°C, TMS, ppm): *δ*_H_ = 4.24–4.02 (m, 2H, CH_2_), 3.81–3.72 (m, 2H, CH_2_), 3.61–3.59 (m, 1H, CH), 2.17 (s, 3H, CH_3_ of acetone), 1.95 (1H, OH), 1.45 (s, 3H, CH_3_), 1.38 (s, 3H, CH_3_). ^13^C NMR (100 MHz, CDCl_3_, 25°C, TMS, ppm): *δ*_C_ = 109.4 (CH_3_CCH_3_), 76.1 (CH), 65.71(CH_2_), 63.0 (CH_2_), 30.9 (CH_3_ of acetone), 26.7 (CH_3_), 25.2 (CH_3_). GC–MS analysis showed a major peak at 5.50 min and a minor peak at 5.84 min (Figure S5), corresponding to solketal and its six‐membered ring isomer, respectively. The reaction catalyzed by RSul100 achieved 99% glycerol conversion and 98% selectivity toward solketal. The catalyst's high selectivity enabled efficient and directed synthesis of solketal, motivating further optimization of the acetalization conditions.

Furthermore, FT‐IR spectra of glycerol and synthesized solketal were obtained (Figure S2). The O─H stretching band in solketal exhibited lower intensity compared to glycerol, due to the presence of only one free hydroxyl group. Additionally, bands at 1370, 1155, 1116, and 1042 cm^−1^ were observed in the solketal spectrum but absent in glycerol, confirming glycerol conversion to solketal. The FT‐IR spectra of the synthesized product matched well with previously reported data [27, 58].

### 3.3 Optimization of Reaction Parameters

**FIGURE 6:**
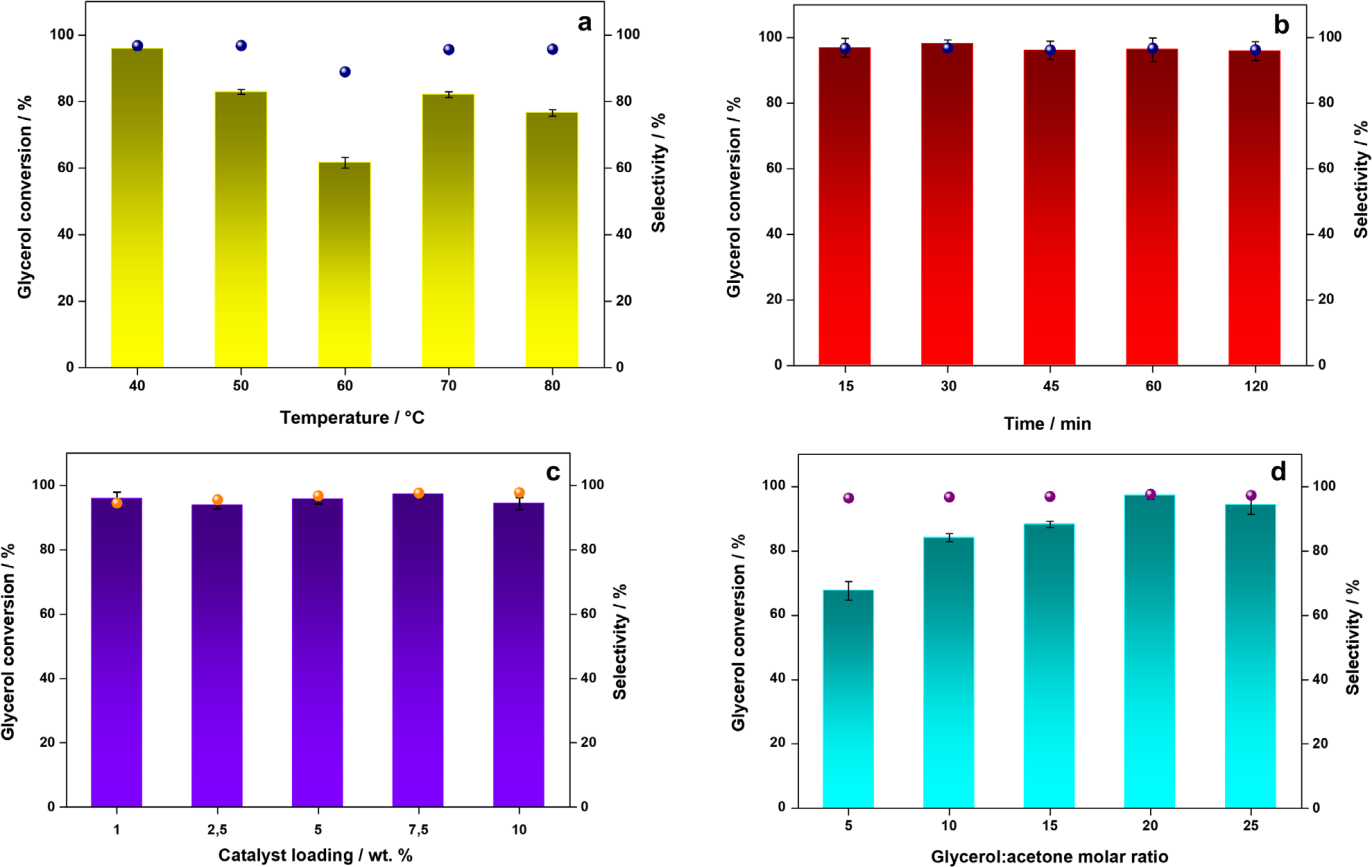
Influence of different parameters on the acetalization of glycerol. (a) Effect of temperature: glycerol to acetone molar ratio 1:20, 5 wt% catalyst, 120 min; (b) effect of reaction time: glycerol to acetone molar ratio 1:20, 5 wt% catalyst, 40°C; (c) effect of catalyst loading: glycerol to acetone molar ratio 1:20, 40°C, 30 min, and (d) effect of glycerol to acetone molar ratio: 7.5 wt% catalyst, 40°C, 30 min. Bars indicate glycerol conversion, and dots indicate selectivity. The values are expressed as mean ± standard deviation (*n* = 3).

Based on the initial results, reaction parameters were optimized to maximize solketal production using the catalyst RSul100. The effects of reaction time, temperature, catalyst loading, and glycerol‐to‐acetone molar ratio were systematically investigated. The results are summarized in Figure 6, which presents the glycerol conversion and the selectivity toward solketal.

The influence of temperature on the acetalization reaction was investigated by conducting experiments at 40°C, 50°C, 60°C, 70°C, and 80°C, using a glycerol‐to‐acetone molar ratio of 1:20, 5 wt% catalyst loading, and a reaction time of 120 min (Figure 6a). The highest glycerol conversion (96%) was achieved at 40°C. Increasing the temperature above 60°C led to a decrease in solketal production, which can be attributed to the exothermic nature of the acetalization reaction, higher temperatures shift the equilibrium toward the reactants, thereby reducing conversion. Additionally, since solketal is a kinetically controlled product, elevated temperatures favor the formation of the thermodynamically more stable six‐membered ring isomer. Thus, 40°C is considered the optimal temperature for solketal synthesis. This behavior is consistent with previous findings reported in the literature [58, 67, 68, 69, 70, 71].

The effect of reaction time was evaluated under optimized temperature conditions (40°C), while maintaining a fixed glycerol to acetone molar ratio of 1:20 and a catalyst loading of 5 wt%. Reaction time was varied from 15 to 120 min (Figure 6b). A rapid increase in glycerol conversion was observed within the first 15 min, followed by a gradual rise, reaching a plateau after 30 min. This behavior indicates that the reaction approached equilibrium after 60 min [72]. However, when selectivity was also taken into account, the highest selectivity toward solketal (97%) was observed at 30 min, with a corresponding glycerol conversion of 96%. Therefore, 30 min was selected as the optimal reaction time, as it ensures both high conversion and selectivity while promoting a more sustainable approach with lower energy demand.

Catalyst loading was evaluated by varying the catalyst mass relative to glycerol (2.5, 5.0, 7.5, and 10 wt%) under the optimized conditions of 40°C, 30 min reaction time, and a glycerol‐to‐acetone molar ratio of 1:20 (Figure 6c). The results showed that glycerol conversion increased with catalyst loading up to 7.5 wt%, at which point maximum efficiency was achieved, with 97% conversion and 98% selectivity toward solketal. At higher catalyst loadings, conversion remained nearly constant, which may indicate the onset of product hydrolysis. Therefore, 7.5 wt% was defined as the optimal catalyst loading, balancing high catalytic performance with efficient material use [58, 73].

The effect of the glycerol to acetone molar ratio was investigated at ratios of 1:5, 1:10, 1:15, 1:20, and 1:25 under the previously optimized conditions (40°C, 7.5 wt% catalyst, and 30 min reaction time) (Figure 6d). Increasing the amount of acetone enhanced glycerol conversion up to a ratio of 1:20, at which 97% conversion and 98% selectivity toward solketal were achieved. Beyond this ratio, no significant improvement was observed, and a slight decrease in yield was noted at 1:25. This may be attributed to the saturation of active sites by acetone, limiting its reactivity with glycerol [58, 74, 75]. While excess acetone shifts the equilibrium toward solketal formation, lower molar ratios favor the reverse reaction, thereby decreasing the overall yield [68]. Furthermore, under conditions of low acetone concentration (i.e., high glycerol content), the elevated viscosity of the reaction mixture impairs proper homogenization of the medium, potentially limiting the overall efficiency of the process. Based on these observations, a molar ratio of 1:20 (glycerol:acetone) was identified as the most suitable for this system [71, 72, 74, 76, 77, 78, 79].

After identifying and optimizing the reaction conditions using the sulfonated catalyst RSul100, a larger‐scale reaction was performed. In this experiment, 72 mmol of glycerol (6.67 g) were reacted with 1.448 × 10^3^ mmol of acetone (106.5 mL) in the presence of 0.500 g of catalyst (7.5% w w^−1^) for 30 min at 40°C. The reaction yielded a glycerol conversion of 96.6% and a solketal selectivity of 97%, demonstrating that the catalyst maintains high efficiency even under increased reaction scale.

### 3.4 Green Parametric Studies on Acetalization of Glycerol

In line with the principles of green chemistry, minimizing waste generation is essential for the development of environmentally benign and sustainable chemical processes. Accordingly, evaluating the environmental impact of the catalytic system is fundamental to assessing its overall sustainability. Therefore, the production of solketal from glycerol catalyzed by RSul100 was assessed using both catalytic performance and green chemistry metrics, namely, turnover frequency (TOF), environmental factor (*E*‐factor), atom economy (AE), and process mass intensity (PMI). Together, these parameters provide a comprehensive evaluation of the efficiency, resource utilization, and environmental compatibility of the proposed process. These metrics were quantified according to Equations (4)–(6) [80].

(4) $$E-factor=\frac{Produced\;waste\;mass\;\left(g\right)}{Produced\;solketal\;mass\;\left(g\right)}$$

(5) $$\begin{array}{ccc} & & Atom\;economy\;\left(AE\right)\\ & & =\frac{Mass\;of\;the\;product\;\left(g\right)}{Total\;mass\;of\;all\;the\;substance\;produced\;\left(g\right)}\times 100\;\% \end{array}$$

(6) $$PMI=\frac{Total\;mass\;used\;in\;acetalization\;process\;\left(g\right)}{Produced\;solketal\;mass\;\left(g\right)}$$

Table S2 presents the detailed calculations of all E‐metric parameters evaluated in this study. The environmental factor (*E*‐factor) represents the ratio between the mass of waste generated and the mass of the desired product and is widely used to assess the environmental performance of chemical processes. Lower *E*‐factor values indicate reduced waste generation and, consequently, a more sustainable process. In this study, an excess of acetone (glycerol:acetone molar ratio of 1:20) was employed to shift the reaction equilibrium toward product formation and achieve an exceptional solketal yield of 99.6%. However, since the reaction stoichiometry requires only one mole of acetone per mole of glycerol, the excess acetone remained unreacted and was fully recovered after the reaction using a rotary evaporator and subsequently reused in further acetalization cycles. Therefore, assuming complete recovery and reuse of the unreacted acetone, the calculated *E*‐factor was 0.140. This remarkably low value indicates minimal waste generation and highlights the excellent environmental performance of the proposed process. A comparison with the literature further emphasizes the sustainability of the present catalytic system. Maurya and Sharma [81] reported an efficient SZZ catalyst that achieved 99.3% glycerol conversion and 98% solketal selectivity; however, the corresponding *E*‐factor was 0.81, substantially higher than the value obtained in this work. This comparison demonstrates that the RSul100 catalyst combines excellent catalytic performance with enhanced material efficiency.

In contrast, when acetone recovery is not considered, the *E*‐factor increases dramatically to 8.83. The approximately 63‐fold increase in the *E*‐factor clearly demonstrates the critical role of solvent recovery and recycling in reducing waste generation and improving the overall sustainability of the process. This observation is fully consistent with the principles of green chemistry, which identify waste prevention, resource efficiency, and solvent recycling as key strategies for the development of environmentally benign chemical processes [82, 83].

AE, however, is defined as the mass of the isolated product relative to the total mass of all products generated during the synthesis process [81]. High AE values indicate a highly efficient and inherently cleaner chemical pathway, maximizing the utilization of raw materials while minimizing the generation of by‐products, with a theoretical maximum value of 100%. The process investigated in this work exhibited an AE of 87.69%, indicating that most of the reactant mass was effectively incorporated into the desired product. This high AE value is inherent to the acetalization reaction, in which glycerol and acetone are efficiently converted into solketal, with water being the only stoichiometric by‐product [17]. Consequently, this result highlights the intrinsic efficiency of the reaction from a green chemistry perspective and demonstrates the effective utilization of resources throughout the process.

PMI is defined as the ratio between the total mass of materials used in the process and the mass of the desired product obtained. As a green chemistry metric, PMI provides a measure of material efficiency, with lower values indicating more sustainable processes and reduced resource consumption [80]. The PMI calculated for the present process was 9.47, indicating that 9.47 g of materials were required to produce 1 g of solketal. This value is primarily influenced by the large excess of acetone employed (glycerol:acetone molar ratio of 1:20), a strategy adopted to shift the reaction equilibrium toward product formation and achieve a glycerol conversion of 99.6%. However, the environmental impact associated with this excess is substantially mitigated by the recovery and reuse of the unreacted acetone. Consequently, although the use of excess acetone increases the overall material input, it enables excellent catalytic performance while maintaining favorable sustainability metrics, particularly when solvent recycling is incorporated into the process design [82, 83].

Overall, the combination of high AE, low *E*‐factor under solvent recovery conditions, and excellent catalytic performance demonstrates that the proposed process constitutes an environmentally attractive strategy for solketal production. These results reinforce the potential of biomass‐derived sulfonated carbons as sustainable catalysts for glycerol valorization and the development of greener catalytic technologies.

### 3.5 Catalyst Recycling

**FIGURE 7:**
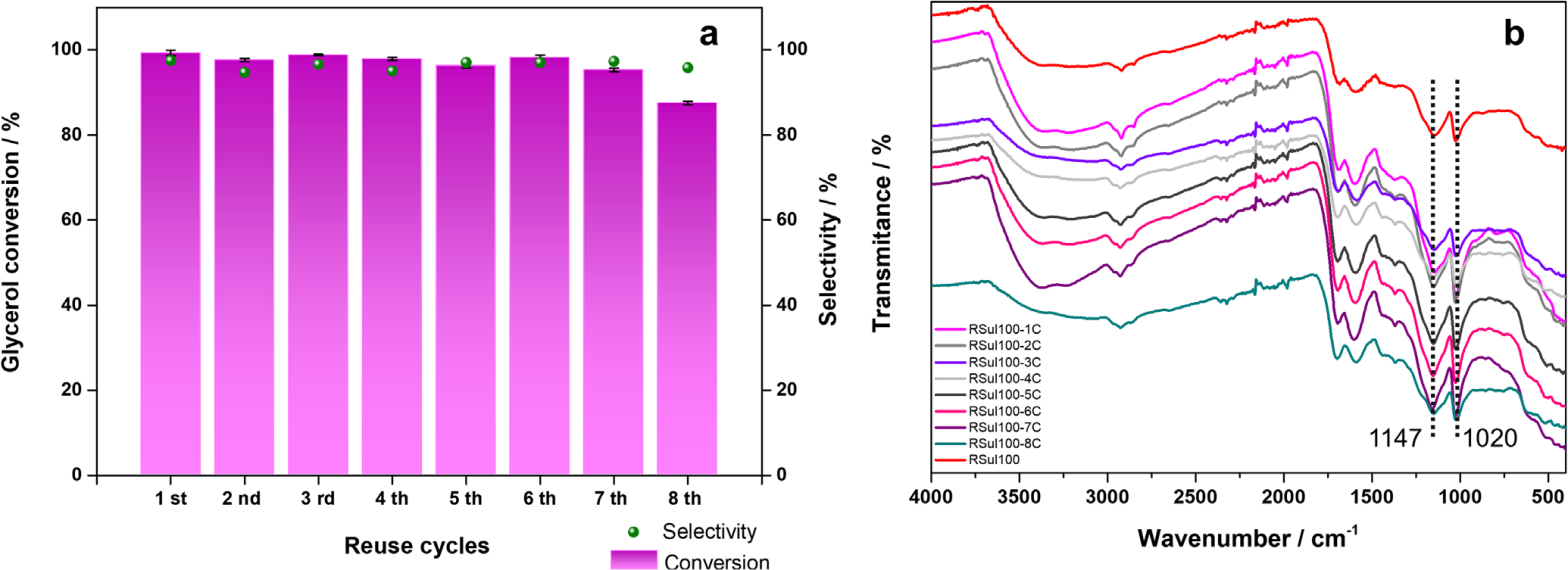
Catalyst recycling study. (a) Glycerol conversion and solketal selectivity during catalyst reuse (40°C, glycerol/acetone molar ratio 1:20, 7.5 wt% catalyst, 30 min) and (b) FT‐IR spectra of RSul100 catalyst after each reuse cycle. Glycerol conversion values are expressed as mean ± standard deviation (*n* = 3).

The reuse of the RSul100 (Figure 7) catalyst was evaluated over eight consecutive cycles under the optimized reaction conditions: 40°C, a glycerol‐to‐acetone molar ratio of 1:20, 7.5 wt% catalyst loading, and a reaction time of 30 min (Figure 7a). After each cycle, the catalyst was recovered by centrifugation, washed four times with ethanol, and dried in an oven at 80°C for 5 h prior to reuse. The recycled catalysts, designated RSul100‐*X*C (where *X* = 1, 2, 3, 4… denote the cycle number), were characterized by FT‐IR (Figure 7b) and EDS (Figure S6 and Table S3).

The RSul100 catalyst exhibits high stability over multiple runs. The results demonstrated that the material maintained high performance during the first six reuse cycles, with glycerol conversions ranging from 97% to 99% and solketal selectivity between 95% and 98%. A slight decrease was observed in the seventh cycle, with the conversion dropping to 95% and the selectivity to 97%. In the eighth cycle, however, a more pronounced decline occurred, with the conversion decreasing to 87% and the selectivity to 96%. Despite this reduction in the final run, the overall durability of RSul100 remains outstanding, as it sustains high catalytic activity for more cycles than most sulfonated carbon‐based catalysts reported in recent literature.

A slight variation in glycerol conversion was observed throughout successive reuse cycles; however, the catalytic activity remained essentially stable for seven of the eight runs [84]. The differences between cycles are likely associated with the gradual deactivation of strong acid sites due to interactions between the ─COOH and ─SO_3_H groups on the catalyst surface and the hydroxyl groups of glycerol [84], which is consistent with the progressive decrease in the intensity of the FT‐IR band corresponding to O─H stretching. Furthermore, the FT‐IR spectra of the reused catalysts, obtained after each cycle, exhibit the characteristic symmetric (1020 cm^−1^) and asymmetric (1147 cm^−1^) S═O stretching bands, further corroborating the structural stability of the sulfonated carbon catalyst [51, 85]. To monitor the composition and potential leaching of the active species, SEM‐EDS analyses were performed cycle by cycle after each individual reuse run (Figure S6 and Table S3). As shown in Table S3, a gradual decrease in surface sulfur content was observed during the initial cycles, dropping from 4.44% in the fresh catalyst to 1.46% by the fourth cycle. This initial loss is commonly attributed to the leaching of weakly bound or physiosorbed sulfonic species formed during the in situ functionalization. Crucially, from the fourth cycle onward, the sulfur content reached a structural stabilization plateau, remaining relatively constant and resilient until the eighth cycle. This chemical stabilization strongly indicates that a significant density of robustly anchored ─SO_3_H groups remains embedded within the carbon matrix, ensuring sustained catalytic efficiency and confirming the true heterogeneous nature of RSul100.

Reusability is a key characteristic for confirming the heterogeneous nature of a catalyst. The stability observed during the initial cycles suggests that the active sites of RSul100 were not significantly leached, retaining catalytic activity throughout at least seven consecutive runs. Overall, these results demonstrate that the RSul100 catalyst exhibits good stability and reusability, reinforcing its potential for sustainable acetalization processes. These findings are consistent with previous studies on sulfonated carbon‐based solid acids, which have also shown satisfactory performance over multiple reaction cycles [86, 87, 88].

### 3.6 Comparison of RSul100 With Other Reported Catalysts

To further evaluate the importance and novelty of the RSul100 catalyst, Table 5 presents a comprehensive comparison between its performance under optimized conditions and other catalysts reported in the literature for glycerol acetalization. When compared with other systems, RSul100 demonstrates significant advantages in terms of energy efficiency and reaction time. For example, while catalysts such as Zr‐PhDPA‐oPA [89], HPW_12_@NiFe_2_O_4_@B2, [90], and Cu‐FSM‐16 [91] require significantly longer reaction times (1440, 180, and 150 min, respectively), RSul100 achieved 97% conversion in just 30 min at a mild temperature of 40°C. Furthermore, while most catalysts, including PCAC‐PhSO_3_H [26], SA‐60 [92], and UiO‐SO_3_H‐30 [93], require higher temperatures (70°C–80°C) for efficient conversion, the proposed catalyst operates effectively at much lower thermal energy levels.

**TABLE 5:** Performance comparison of RSul100 catalyst with reported catalysts for solketal synthesis.

Catalyst	Temperature (°C)	Time (min)	Molar ratio	Reuse cycles	Conversion (%)	Selectivity (%)	Reference
PCAC‐PhSO_3_H (Sulfonated pine cone activated carbon)	80	20	1:5	6	98	93	Das et al. [26]
SA‐60 (Spherical SiO_2‐_Al_2_O_3_)	80	80	1:10	5	94	97	Wang et al. [92]
HPW_12_@NiFe_2_O_4_@B2 (Magnetic Polyoxometalate@Biochar)	60	180	1:15	4	89	96	Pellaumail et al. [90]
Zr‐PhDPA‐oPA (Zirconium (IV) layered phosphonate‐phosphate)	50	1440	1:15	5	85	98	Ghanemnia et al. [89]
Cu‐FSM‐16 (Copper loaded mesoporous silica)	60	150	1:2	4	85	90	Singh & Gosu [91]
UiO‐SO_3_H‐30 (Sulfated Zr‐based MOF)	60	90	1:14	5	79	99	Wang & Gong [93]
SO_3_H‐HC‐*Ma*L‐B (Porous sulfonated biochar)	70	60	1:5	6	98	97	D. et al. [80]
rGO‐SO_3_H (Sulfonated reduced graphene oxide)	50	60	1:12	4	98	98	Gil‐Gavilán et al. [95]
FeCl_3_/γ‐Al_2_O_3_‐IPA (Porous spherical alumina)	25	30	1:10	6	99	98	Zhang et al. [94]
RSul100	40	30	1:20	8	97	98	Present work

In addition, this study reports a selectivity of 98% toward solketal, a value that exceeds most previously documented systems, such as Cu‐FSM‐16 (90%) and PCAC‐PhSO_3_H (93%) [26, 91]. Although the UiO‐SO_3_H‐30 [80] catalyst exhibits a slightly higher selectivity (99%) than RSul100, it requires a threefold longer reaction time (90 min vs. 30 min) to achieve high conversion. This comparison highlights that RSul100 offers an optimal balance between high selectivity and mild conditions.

A crucial highlight is the catalyst's stability; RSul100 remained efficient for 8 reuse cycles, exceeding the reusability reported for all other catalysts in this comparison, which typically range from 4 to 6 cycles. Even the FeCl_3_/γ‐Al_2_O_3_‐IPA [94] catalyst, the most efficient among those reported, achieving 99% conversion at room temperature within 30 min, could only be reused for 6 cycles. This fact underscores the superior performance of RSul100 in terms of catalyst heterogeneity and structural integrity. Even after the eighth cycle, glycerol conversion remained high at approximately 90%. This superior performance under mild conditions (40°C and 30 min), combined with exceptional reusability, underscores the novelty and potential of RSul100 for more sustainable glycerol acetalization processes.

### 3.7 Possible Reaction Mechanism

**SCHEME 3:**
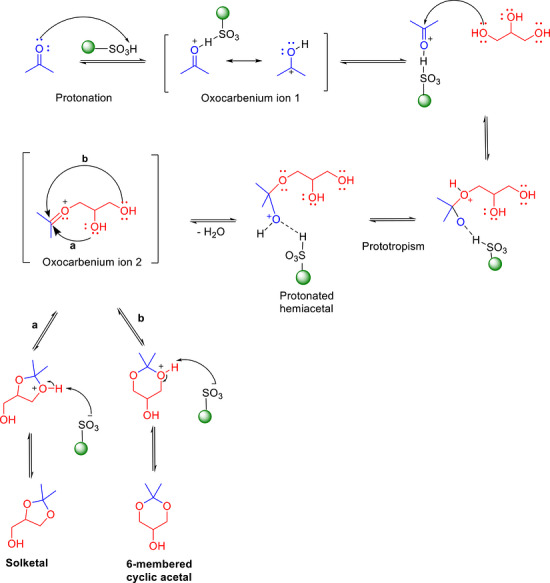
The proposed mechanism for glycerol acetalization reaction with acetone.

According to the literature [58, 96, 97], the acetalization of glycerol with acetone typically occurs via the acid‐catalyzed activation of the carbonyl group, followed by a nucleophilic attack by glycerol. Considering the acidic properties of the catalyst employed herein, a similar mechanism is proposed. As illustrated in Scheme 3, the reaction is initiated by the protonation of the carbonyl group of acetone by the Brønsted acid sites present on the catalyst surface (─SO_3_H), leading to the formation of an activated oxocarbenium ion intermediate (**1**) [58, 96, 97]. This intermediate is highly susceptible to nucleophilic attack by one of the hydroxyl groups of glycerol, resulting in the formation of a protonated hemiacetal intermediate. The subsequent elimination of water gives rise to a second oxocarbenium ion, which undergoes intramolecular cyclization. This critical step leads to the formation of two distinct cyclic acetal products: a five‐membered ring (a) and a six‐membered ring (b), with the former widely known as solketal. The predominance of solketal is generally attributed to its higher thermodynamic stability, reduced conformational strain, and kinetically favored formation pathway compared to the corresponding six‐membered cyclic acetal, characteristics commonly associated with five‐membered rings in such systems.

In addition to promoting the initial protonation of acetone, the ─SO_3_H groups play a fundamental role throughout the catalytic cycle by stabilizing the positively charged intermediates and facilitating both the dehydration of the hemiacetal intermediate and the subsequent cyclization step. The strong Brønsted acidity provided by these surface functionalities enhances carbonyl activation and decreases the activation energy barrier associated with the formation of the cyclic acetals, thereby boosting both glycerol conversion and solketal selectivity. Furthermore, the high density of accessible acid sites on the catalyst surface promotes efficient interactions between the reactants and the active centers, directly contributing to the superior catalytic performance observed for RSul100 [25, 26, 76].

## 4 Conclusion

In this study, a sulfonated carbon catalyst (RSul100) was successfully synthesized from lavender biomass residue (obtained after essential oil extraction) via HTC with in situ sulfonation. Structural and spectroscopic characterizations (XRD and FT‐IR ) confirmed the amorphous nature of the carbon matrix and the effective incorporation of ─SO_3_H functional groups. The catalyst demonstrated excellent activity and selectivity in the acetalization of glycerol with acetone under mild conditions, achieving up to 97% conversion and 98% selectivity toward solketal. Reaction optimization revealed that 40°C, 30 min, 7.5 wt% catalyst loading, and a 1:20 glycerol to acetone molar ratio provided the most efficient and sustainable conditions. A key advantage of the proposed system is its high efficiency under exceptionally mild conditions (40°C and 30 min), offering a significant reduction in energy demand and processing time when compared to benchmark catalysts in the field.

Notably, the RSul100 catalyst demonstrated outstanding stability and reusability compared to recent literature reports. It maintained high catalytic performance for up to 8 reuse cycles, surpassing similar systems that typically show a decrease in activity after 4–6 cycles, while preserving over 96% conversion and 97% selectivity through seven consecutive runs. These results underscore the potential of RSul100 for application in green chemical processes and highlight the value of agro‐industrial waste valorization for developing efficient, low‐cost, and environmentally benign heterogeneous catalysts.

Overall, the findings demonstrate that RSul100 is a promising candidate for biomass‐derived catalytic systems, offering a viable route for glycerol upgrading and contributing to the advancement of circular and sustainable chemical processes. Moreover, the use of this residue can be important for adding value to the lavender essential oil production chain.

## Author Contributions

**Patrícia F. Pinheiro**: conceptualization, resources, supervision, project administration. **Gabriel H. M. de O. Pinto**: methodology. **Guilherme R. Pereira**: investigation, methodology. **Eraldo Lima**: methodology. **Kelly R. Francisco**: methodology, writing – review and editing, supervision. **Garbas A. dos S. Junior**: formal analysis, methodology, conceptualization. **Julianne A. Bruno**: conceptualization, funding acquisition, methodology, formal analysis, writing – review and editing, investigation, writing – original draft, data curation, resources. **Ulisses A. Pereira**: writing – review and editing, methodology.

## Conflicts of Interest

The authors declare no conflicts of interest.

## Supporting information




**Supporting File 1**: cbdv71558‐sup‐0001‐SuppMat.docx.

## Data Availability

The data that supports the findings of this study are available in the supplementary material of this article.
