# Supplementary material for: Efficient Solketal Synthesis via Acetalization Using Sulfonated Carbon From Lavender Leaf Waste After Essential Oil Extraction
Source: Chem Biodivers. 2026 Aug 8;23(8):e71558. doi: 10.1002/cbdv.71558 (PMC13452547; doi:10.1002/cbdv.71558)
Supplement: Supplementary file 1 [file CBDV-23-e71558-s001.docx]

**Supporting Information**

Efficient Solketal Synthesis via Acetalization Using Sulfonated Carbon from Lavender Leaf Waste After Essential Oil Extraction

Julianne A. Bruno,^[a]^ Guilherme R. Pereira,^[a]^ Kelly Roberta F. M. de Paula,^[b]^ Gabriel Henrique M. de O. Pinto,^[a]^ Eraldo R. de Lima,^[c]^ Ulisses Alves Pereira,^[d]^ Garbas A. dos S. Junior,^[a]^ Patrícia Fontes Pinheiro*^[a]^

Dedication

[a]	J. A. Bruno, G. R. Pereira, G. H. M. de O. Pinto, E. R. de Lima, G. A. dos S. Junior, P. F. Pinheiro

Department of Chemistry
Federal University of Viçosa
Avenida Peter Henry Rolfs, s/n Campus Universitário, 36570-900, Viçosa, MG, Brazil.
E-mail: [patricia.pinheiro@ufv.br](mailto:patricia.pinheiro@ufv.br).

[b]	K. R. F. M. de Paula
Department of Natural Sciences, Mathematics and Education
Federal University of São Carlos
Rodovia Anhanguera km 174, 13600-970, Araras-SP, Brazi

[c] E. R. de Lima

Department of Entomology - BIOAGRO

Federal University of Viçosa
Avenida Peter Henry Rolfs, s/n Campus Universitário, 36570-900, Viçosa, MG, Brazil.

[d]	U. A. Pereira

Institute of Agricultural Sciences

Federal University of Minas Gerais

Avenida Universitária 1000, Bairro Universitário, CEP 39404-547, Montes Claros, MG, Brazil.

**Abstract:** Biomass-based catalyst research is advantageous for the biodiesel industry's sustainability due to the catalysts' readily available raw materials, non-toxic properties, and biodegradability. In this study, sulfonated carbon catalysts were synthesized from lavender biomass residue, obtained after essential oil extraction, through hydrothermal carbonization followed by in situ sulfonation, and applied in the acetalization of glycerol with acetone to produce solketal, a bio-based fuel additive. To the best of our knowledge, this type of residue has not yet been explored for this specific application. Characterization by XRD, FT-IR, NMR, and GC–MS confirmed the amorphous carbon structure and successful incorporation of –SO₃H groups. The catalyst exhibited excellent performance under mild conditions, achieving 97% glycerol conversion and 98% selectivity toward solketal. Optimal conditions (40 °C, 30 min, 7.5 wt% catalyst loading, and a glycerol to acetone molar ratio of 1:20) afforded high glycerol conversion and selectivity toward solketal. The catalyst exhibited remarkable stability, maintaining >96% conversion and >97% selectivity after seven reuse cycles, confirming the potential of lavender waste as a sustainable precursor for catalyst development. These results highlight the potential of lavender agro-residues as a sustainable carbon source for the production of efficient solid acid catalysts in renewable fuel applications.

**Supporting Information**

Screening of Catalysts for the Production of Solketal from Glycerol^……..……………………………………………………..……..…^**Figure S1**

FT-IR spectra of glycerol used in comparison to solketal obtained by sulfonated lavender residue

Catalyst...^……………………………………………………………………………………………………………………………………………………………………………^**Figure S2**

Textural properties of the materials derived from lavender residues, determined by nitrogen adsorption–desorption analysis^……………………………………………………………………………………………………………………………………………….……^ **Figure S3 and Table S1**

NMR data for the product of glycerol acetalization by sulfonated lavender residue catalyst^…………………………..….^**Figure S4**

Total ion chromatogram of the synthesized solketal and associated mass spectrum, supporting the identification and structural assignment of the product^……………………………………………………………………………………...…………………….……………...…^**Figure S5**

Green Parametric Studies on Acetalization of Glycerol^………………………………………………………………………………………….……^**Table S2**

Energy-dispersive X-ray spectroscopy (EDS) profiles of the catalyst after successive reuse cycles, evidencing the evolution of surface elemental composition.^…………………………………………………………………………………………………………………………………………………………..…......……^**Figure S6**

Relative mass percentage of carbon (C), oxygen (O) and sulfur (S) in the recycled catalysts^……………………………………………………………………………………………………………………………………………………………………..…………^**Table S3**

**Screening of Catalysts for the Production of Solketal from Glycerol**

**Figure S1.** Conversion of (a) sulfonated and (b) non-sulfonated catalysts, and selectivity of (c) sulfonated and (d) non-sulfonated catalysts in the acetalization of glycerol. Reactions were carried out at room temperature for 6 hours with a glycerol: acetone molar ratio of 1:20 and 5 wt% catalyst loading

**FT-IR spectra of glycerol used in comparison to solketal obtained by sulfonated lavender residue catalyst**

**Figure S2.** FT-IR spectrum of (a) glycerol and (b) solketal product.

**Textural properties of the materials derived from lavender residues, determined by nitrogen adsorption–desorption analysis.**

**Figure S3**. Nitrogen adsorption–desorption isotherms and pore size distribution curves of the materials derived from lavender residues. The inset graphs correspond to the pore size distribution obtained from the BJH method.

**Table S1**. Analysis of the surface properties of materials obtained from lavender residue.

| Catalyst | SBET (dm^2^/g) | Pore diameter (nm) | Pore volume (10^-4^ cm^3^/g) |
| --- | --- | --- | --- |
| RSul100 | 1.7 | 6.55 | 4.2 |
| RSul130 | 35.6 | 4.89 | 3.6 |
| RBr100 | 0.0 | 31.9 | 4.0 |
| RHCl | 0.0 | 31.7 | 1.9 |
| RNat | 0.0 | 32.1 | 4.6 |

**NMR data for the product of glycerol acetalization by sulfonated lavender residue catalyst**

^1^H NMR (400 MHz, CDCl_3_, 25 °C, TMS, ppm): *δ*_H_ = 4.24-4.02 (m, 2H, CH_2_), 3.81-3.72 (m, 2H, CH_2_), 3.61-3.59 (m, 1H, CH), 2.17 (s, 3H, CH_3_ of acetone), 1.95 (1H, OH), 1.45 (s, 3H, CH_3_), 1.38 (s, 3H, CH_3_).

^13^C NMR (100 MHz, CDCl_3_, 25 °C, TMS, ppm): *δ*_C_ = 109.4 (CH_3_CCH_3_), 76.1 (CH), 65.71(CH_2_), 63.0 (CH_2_), 30.9 (CH_3_ of acetone), 26.7 (CH_3_), 25.2 (CH_3_).

 **Figure S4.** (a) ^1^H NMR and (b) ^13^C NMR of the product

**Total ion chromatogram of the synthesized solketal and associated mass spectrum, supporting the identification and structural assignment of the product.**

EM, m/z (%): 132 (C_6_H_12_O_3_); 117 (37), 101 (25), 73 (6), 57 (23), 43 (100)

**Figure S5.** (a) GC data for synthesized solketal; (b) MS spectra of solketal and (c) MS spectra of solketal obtained from the instrument's reference library

**Green Parametric Studies on Acetalization of Glycerol**

**Table S2**. E-metrics calculation

| **Given data** | | |
| --- | --- | --- |
| Glycerol taken |  | 6.67 g |
| Molar mass of glycerol | 92.09 g/mol |  |
| Moles of glycerol | 6.67 ÷ 92.09 | 0.0724 mol |
| Molar ratio (Glycerol: Acetone) | 1:20 |  |
| Molar mass of acetone | 58.08 g/mol |  |
| Moles of acetone | 20 × 0.0724 mol | 1.448 mol |
| Mass of acetone | 1.448 × 58.08 | 84.09 g |
| Yield of solketal | 99.6% |  |
| Molar mass of solketal | 132.16 g/mol |  |
| Catalyst (7.5 wt.% of glycerol) | 0.075 × 6.67 | 0.500 g |
| **Solketal Produced**  Moles of glycerol reacted = 0.0724 mol  99.6% yield → Moles of solketal = 0.0724 × 0.996 = 0.0721 mol  Mass of solketal = 0.0721 × 132.16 = 9.53 g | | |
| **Water Produced (by-product)**  Water: 1 mol formed per mol of solketal  Moles of water = 0.0721 mol  Mass = 0.0721 × 18.02 = 1.29 g | | |
| **Unreacted Acetone**  Total acetone used = 1.448 mol  Acetone reacted = 0.0721 mol  → Unreacted acetone = 1.448 - 0.0721 = 1.3759 mol  Mass = 1.3759 × 58.08 = 79.91 g (**recovered)** | | |
| ***E-metrics calculation***   - $E-factor = \frac{Produced waste mass (g)}{Produced solketal mass (g)}$   = $\frac{1.29}{9.19}$ = 0.140 (considering acetone recovery)  = $\frac{1.29 +79.91}{9.19}$ = 8.83 (if acetone recovery is not considered   - $Atom economy = \frac{Mass of the product (g)}{Total mass of all the substance produced (g)}\times100 \%$   = $\frac{9.19}{9.19 +1.29} \times100 \%$ = 87.69%   - $PMI = \frac{Total mass used in acetalization process \left( g \right)}{Produced solketal mass \left( g \right)}$   = $\frac{6.67 + 79.91 + 0.500}{9.19}$= 9.47 | | |

**Energy-dispersive X-ray spectroscopy (EDS) profiles of the catalyst after successive reuse cycles, evidencing the evolution of surface elemental composition.**

**Figure S6.** EDS spectra of the recycled catalysts recovered after each cycle: RSul100-1st, RSul100-2st, RSul100-3st, RSul100-4st, RSul100-5st, RSul100-6st, RSul100-7st and RSul100-8st

**Table S3.** Relative mass percentage of carbon (C), oxygen (O) and sulfur (S) in the recycled catalysts ^[a]^.

| Sample | C (%) | O (%) | S (%) |
| --- | --- | --- | --- |
| RSul100 | 60.09 | 35.48 | 4.44 |
| RSul100-1st | 61.47 | 34.32 | 4.21 |
| RSul100-2st | 59.71 | 35.56 | 2.73 |
| RSul100-3st | 64.40 | 33.64 | 1.96 |
| RSul100-4st | 63.48 | 35.06 | 1.46 |
| RSul100-5st | 63.38 | 35.59 | 1.04 |
| RSul100-6st | 65.03 | 33.30 | 1.67 |
| RSul100-7st | 62.98 | 36.16 | 0.86 |
| RSul100-8st | 63.92 | 35.59 | 0.49 |

[a] SEM-EDS provides semi-quantitative results as it probes only localized regions of the sample.
